# Examining Psychosocial Factors and Community Mitigation Practices to Limit the Spread of COVID-19: Evidence from Nigeria

**DOI:** 10.3390/healthcare10030585

**Published:** 2022-03-21

**Authors:** Ekundayo Shittu, Funmilayo Adewumi, Nkemdilim Ene, Somto Chloe Keluo-Udeke, Chizoba Wonodi

**Affiliations:** 1 Department of Engineering Management and Systems Engineering, School of Engineering and Applied Science, George Washington University, Washington, DC 20052, USA; 2 Women Advocates for Vaccine Access (WAVA), 15 Amazon Street, Off Alvan Ikoku Way, Maitama, Abuja 904101, Nigeria; funmilayoadewumi@wavang.org (F.A.); somtoudeke@wavang.org (S.C.K.-U.); 3 Preston Associates for International Development, Plot 1854 Mahathir Mohammed Street, Off TY Danjuma Street, Asokoro, Abuja 900100, Nigeria; n.ene@prestonassociate.com; 4 Department of International Health, International Vaccine Access Center, Johns Hopkins Bloomberg School of Public Health Baltimore, Baltimore, MD 21205, USA; cwonodi1@jhu.edu

**Keywords:** non-pharmaceutical intervention (NPI), COVID-19, mitigation practices, Nigeria, health policy

## Abstract

We examine the psychosocial factors influencing community adoption of non-pharmaceutical interventions (NPI) to limit the spread of COVID-19. Using data from 990 respondents in communities across Nigeria, we examine the correlation of health behaviors and socioeconomic indicators. We conduct logistic regression to estimate the relationship between mask wearing as a health-seeking NPI with demographic and socioeconomic variables. We estimate separate models in the sensitivity robustness checks with other NPIs and control for differences across sex, age, education, number in household, and the presence of a student in the respondent’s household. A crucial finding is that health-seeking NPI behaviors are statistically significantly affected in different ways by the menu of socioeconomic indicators. The control for age, sex, education, and household size indicates that there is intersectionality of how these factors influence specific mitigation practices. We find that women are more likely to engage in mask wearing, hand washing, and use of hand sanitizers and tissues than men, and the provision of palliatives and access to family supplies significantly enhances community mitigation. Palliatives and access to family supplies enhance most health-seeking behaviors. The implication for pandemic mitigation policy is that minimizing incidence rates requires having responsive initiatives such as information updates on pandemic progression.

## 1. Introduction

At the peak of the coronavirus disease 2019 (COVID-19) pandemic, caused by severe acute respiratory syndrome coronavirus 2 (SARS-CoV-2), in 2020–2021, public health measures were imposed in most countries to limit the spread of the virus, causing significant behavioral changes in human lives across the globe. These measures included non-pharmaceutical interventions (NPIs) that have been classified in four broad categories (personal protective, environmental, social distancing, and travel-related measures) by the World Health Organization (WHO, Geneva, Switzerland) [1]. Personal protective measures include the practice of hand hygiene and respiratory etiquette, and the use of face masks. Environmental measures comprise surface and object cleaning, ultraviolet light, increased ventilation and modifying humidity. Social distancing measures cover contact tracing, isolation, quarantine, school and workplace closures, and avoiding crowding. Travel-related measures include travel advice, restrictions, and border closures. Nigeria, like the rest of the world, implemented these measures to varying degrees of success.

Community mitigation refers to measures designed to be used by individuals, organizations, and governments to reduce the transmission of COVID-19 in the community. NPIs are central to community mitigation strategies, and they are critical in pandemic response plans given the considerable amount of time required to develop and deploy efficacious vaccines and drugs [2]. Their potential impacts in pandemic settings are to delay the introduction, impede the height of the epidemic peak, limit community transmission, and reduce the total number of infections [1]. Community mitigation strategies that also include multilayered NPIs, including handwashing and social distancing, and a combination of government support, protection, and restrictions, are also important in slowing the spread of viral epidemics while awaiting safe and effective vaccines [3]. One of the key messages is that most individuals, irrespective of background, including general populations, students, and athletes, were affected by the pandemic, psychologically, physiologically, and physically. For example, the pandemic had impacts such as altered sleep and circadian rhythms [4,5] and training activities [6] across the world. Other community mitigation strategies include the cancellation or suspension of events, changes to funeral services, and clear communication from health authorities on how to avoid fake news, rumors, and panic [4]. In response to the COVID-19 pandemic, many countries and regions around the world have implemented a wide range of NPIs in varying degrees to limit the spread of the disease [7]. Evidence from around the world has shown that a combination of NPIs and community mitigation measures showed great promise in preventing new cases and controlling the spread of COVID-19 [8,9,10,11,12,13].

To a large extent, the effectiveness of these NPIs involved partnerships that build on community strengths and limit inequities in accessibility [14]. However, the risk perception towards the embrace of these interventions varies in the community. For example, gender plays a vital role in determining the level of risk perception that in turn determines the acceptance of NPIs or other coping strategies [15]. Similarly, the awareness of the existence of morbidity such as diabetes [16], obesity [17], or high blood pressure [18] also influences the severity of the pandemic, either through the incidence, prevalence, or the mortality rates. On the premise that the epidemiology of COVID-19 is not different from that of other diseases in terms of gender susceptibility, evidence from several countries suggested that mortality from COVID-19 infection is higher in men than women, implying that males have a higher likelihood of SARS-CoV-2 susceptibility [19].

The impact of the COVID-19 pandemic on lives and livelihoods in Nigeria led to a range of government responses, particularly in the campaign for NPIs. While vaccines may help the control of COVID-19, Nigeria, like other low- and middle-income countries is unlikely to reach the vaccination coverage rates necessary to combat the disease. Thus, NPIs will continue to play an important part in COVID-19 control, especially in the context of viral variants of concern and vaccine hesitancy. Since the first case of COVID-19 was reported in Nigeria in February 2020, the country, just like many countries around the world, encouraged the practice of NPIs such as regular hand washing, use of face masks, use of hand sanitizers, and physical distancing measures to limit the spread of the disease [20,21]. The level of adherence to the NPIs was low at the community level, particularly towards the end of the first wave of the pandemic. In a series of nationwide biweekly pulse surveys conducted between August and December 2020 on citizens’ experience with the pandemic across Nigeria [21,22], only 32% of respondents reported that the COVID-19 preventive guidelines, including NPIs, were strictly observed in their communities, and 13% stated that the guidelines were not being observed at all. Within the same period, 69% of the respondents noted that community members wore their face masks to cover their noses and mouths, 7% observed masks being worn to cover only their mouths, and 23% noted people wore masks on their chins.

A scan of the national COVID-19 measures and response structures in Nigeria [20,21,22] showed that during the pandemic, in March 2020, a Presidential Task Force (PTF) on COVID-19 was constituted and inaugurated by the President of the Federal Government of Nigeria with similar structures being replicated at state levels. The state task forces work closely with the PTF to adapt and enforce NPIs and stringent community mitigation measures at the state level. These measures included state border shutdowns, interstate travel bans, lockdown measures, curfews and movement restrictions, changes in market operations, school closures, and a limit on mass and social gathering [20,21,22]. The scan also indicated that as of December 2020, while there was a closure of schools and ban on interstate travel in all states, 90% of states changed their market operations, 89% enforced lockdown of nonessential services in the states, and 87% enforced curfews. While the specific duration of lockdown varied among states, it ranged from as long as four months in most states to as short as less than one month in Kogi state [20].

The central hypothesis of this paper is underscored by seeking answers to three interrelated questions. First, what factors affect community adoption of NPIs? Second, what is the correlation between health-seeking behaviors and key socioeconomic indicators? Third, how do we inform pandemic mitigation policies? Understanding the drivers of NPI adoption can help inform strategies to improve the uptake and adherence to NPIs.

This study is conducted in the context of an intervention to enhance Civil Society Organizations’ (CSOs) involvement in the COVID-19 response in Nigeria through improved coordination, capability, communication, and accountability. Thus, it is crucial to understand the factors that affect community adoption of NPIs. This understanding entails knowing the extent of the correlations between health-seeking behaviors and socioeconomic activities. This knowledge could be instrumental in the crafting of pandemic mitigation policies.

## 2. Theoretical Framework

This paper adopted a behavioral model of increasing vaccination to examine demographic and psychosocial factors that could influence the uptake of NPI. This adoption is based on the premise that behavioral interventions that are grounded on a cogent theory are more likely to be effective. The uptake of vaccines where available and affordable is often predicated on three general propositions: (i) the decision to receive vaccination may be motivated by thoughts and feelings; (ii) contemporary social processes such as free riding, i.e., taking advantage of the protection induced by herd immunity could influence vaccination uptake; (iii) facilitated interventions enhanced with reminders and prompts could increase responsiveness [23]. Thus, this paper draws a parallel between the psychological influence on vaccination decisions and community engagement with NPIs.

**Figure 1:**
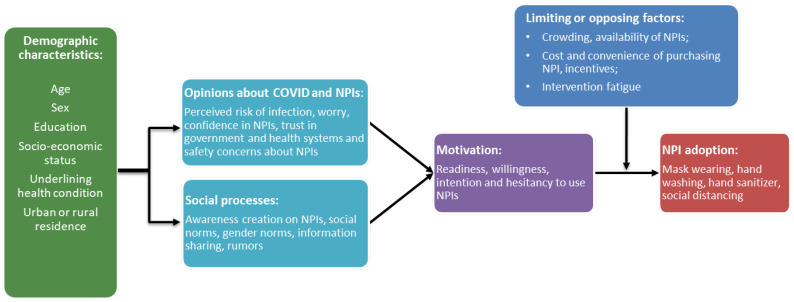
Theoretical framework: The COVID-19 prevention NPIs.

Thus, considering the above, this paper is conducted in the context of Figure 1. With the information on several background characteristics such as sex, age, education, household size, number of students in the household, and accessibility to food and supplies, the premise of the propositions is bifurcated along two tranches: (i) Opinions about COVID-19 and NPIs including the risk perception of infection, worry, confidence in NPIs, trust in the government and health systems, and safety concerns about NPIs, e.g., awareness of the COVID-19 incidence and mortality rates and if they had anyone in their household with a pre-existing diabetes diagnosis. 

By extension, receiving palliatives from the government builds trust in the government; (ii) social processes including awareness creation on NPIs, social norms, gender norms, information sharing, and rumors. For example, accessibility to health facilities implies that people may hear more, and believe more, about NPIs in clinics.

The two propositions combine to motivate the community through increased readiness or higher willingness (or perhaps hesitancy) to use NPIs. It is in the nexus of the motivation and practical issues including crowding, availability of the NPIs, cost and convenience of practicing NPIs, incentives and palliatives, and the extent of intervention fatigue that combine to inform NPI adoption. We note that the motivations, propositions, and practical issues have different effects on different NPIs in the presence of other demographic differences and socioeconomic realities.

## 3. Methods and Materials

This section describes the data collection process, the study areas and population, the variables, and the estimation method employed.

### 3.1. Data and Data Collection

**Figure 2:**
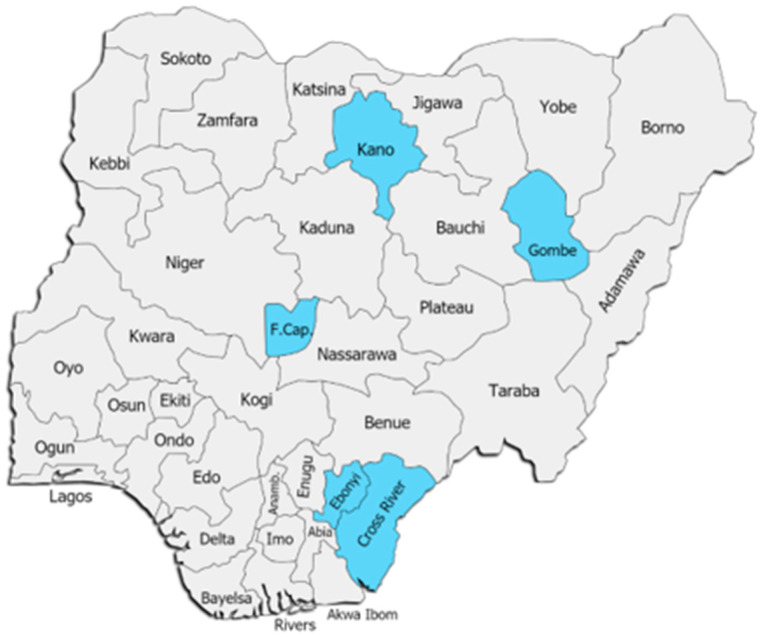
Map of Nigeria showing the states where the communities covered are located.

The cross-sectional data set of observations was based on the survey of 990 respondents administered by the Coordinating and Mobilizing Civil Society Response in Nigeria (COMCiRIN) across communities in four states and the FCT in Nigeria between 3 November and 11 December 2020. The data collection was limited to the states in Nigeria in Figure 2. Ethical approvals were granted by the Health Research Ethics Committees in the FCT, Cross River and Gombe states. Approval was also obtained from the State Primary Health Care Development Agency (SPHCDA) of Ebonyi and Kano states. We confirm that all methods were performed in accordance with the relevant guidelines and regulations. Data were collected by trained surveyors and supervised by trained CSOs in each state. Data collection was conducted via Open Data Kit (ODK) software to eliminate the tedium associated with paper-based surveys. We elicited respondents’ consent by including a brief consent note in the ODK questionnaire. We requested consent. Respondents who declined consent were not included in the survey. Thus, informed consent was obtained from all the participants.

#### 3.1.1. Study Area

The community small sample survey was conducted in three selected local government areas (LGAs) and six communities in each of the five selected states—Cross River, Ebonyi, Gombe, Kano, and the FCT. The states were selected because of the COVID-19 high burden and to have a representation of the six geopolitical zones in the country. The LGAs and communities were randomly selected using Microsoft Excel and R software. To select the three LGAs per state, all the LGAs in each state were listed alphabetically and numbered, after which the three LGAs were selected using the random sample function in Microsoft Excel. The communities were also randomly selected using the probability proportional to sample size (PPS) methodology and the World Health Organization master list of communities in each of the states as sampling frame. The PPS methodology was selected because it ensures that every community in a selected LGA had an equal probability of being selected regardless of community size. This was important because the communities in the LGAs vary widely in size. Security-compromised communities were filtered out of the master list to avoid the risk of security challenges during data collection.

#### 3.1.2. Sample Population

The survey was conducted among community leaders and community members. In each community, ten adults and one community leader were surveyed. This yielded a sample of 66 respondents per LGA and a total sample size 990 for the five states. While community leaders were purposely selected for the survey, community members were randomly selected using the Lots Quality Assessment Survey (LQAS) field methodology. The study population was made up of 990 respondents with distributions as follows: 62% males and 38% females; 57% were aged 50 years and above; 21.7% were uneducated; occupations ranged from farmers, health workers, business owners, civil servants, engineers, and teachers to the retired.

**Figure 3:**
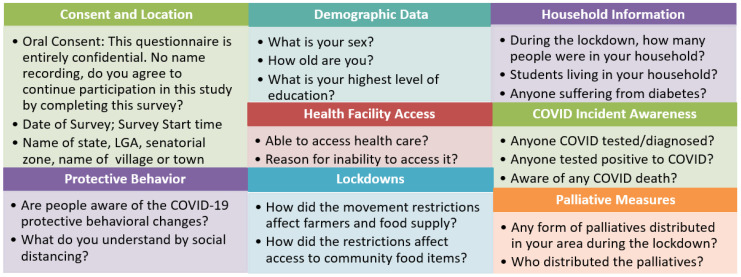
Some of the prompts in the data collection survey.

Figure 3 provides a pictorial representation of the data collection prompts distributed by categories. These categories cover consent, demographic information, location, information on health-seeking or protective behavior, awareness of the COVID-19 related incidence of either diagnosis or death, palliative measures, household information and accessibility to healthcare and other essential supplies such as food stock. The formats of the questions were presented as multiple choice with options including specific values (e.g., level of education) or a range (e.g., age bracket). Each were quantified by the count of responses provided by category.

### 3.2. Dependent Variables

**Figure 4:**
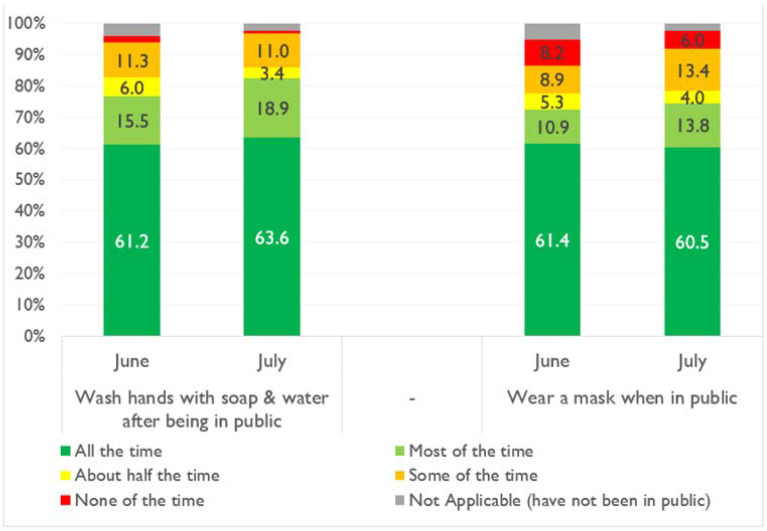
Safe practices in Nigeria from a World Bank study [24].

The dependent variables in this study are the NPIs with the primary one being the respondents’ mask wearing behavior. In the post hoc robustness test, other NPIs are examined. Mask wearing, like other NPIs, is a dichotomous variable with 1 indicating that the respondent engages in mask wearing as a health-seeking NPI behavior and zero otherwise. A recent World Bank study that tracked the socioeconomic impacts of the pandemic in Nigeria shows the prevalence of mask wearing and hand washing in the country from a national longitudinal telephone survey [24]. Figure 4 shows the prevalence of these practices in June and July 2020.

### 3.3. Independent Variables

The independent variables are strictly variables that inform or inhibit the adoption of the health-seeking NPIs. These variables underline the core influential parameters that are in the socioeconomic and other macroeconomic interventions that were prescribed to limit the spread of the disease such as the restriction of movement. Awareness of COVID incidence is a dichotomous variable (i.e., respondent is aware = 1, otherwise = 0). We examined the presence of the awareness of pre-existing morbidity, specifically having someone with diabetes in the household, with aid of the variable diabetes in household (i.e., presence of a diabetic in the household = 1, otherwise = 0). We conjecture that access to a health facility encourages NPIs and capture this variable as health access (i.e., respondent has access = 1, otherwise = 0). In a similar manner, (i) access to palliatives = 1, otherwise = 0; (ii) access to food and essential supplies = 1, otherwise = 0. 

### 3.4. Control Variables

We controlled for the socioeconomic and demographic characteristics that may influence an individual’s health-seeking NPI adoption. The variable sex captures the acknowledged gender of the respondent (i.e., male = 1, female = 0). Age is a non-dichotomous variable just as the variable household size that captures the number of people living in the respondent’s household. We coded the respondent’s education (i.e., educated = 1, otherwise = 0) and the variable student in household (i.e., presence of students in the household = 1, otherwise = 0). 

### 3.5. Estimation Method

We conducted logistic regression to estimate the relationship between mask wearing as a health-seeking NPI and several demographic and socioeconomic variables. We estimated separate models in the sensitivity robustness checks with other NPIs. We controlled for differences across sex, age, education, number in household, and the presence of a student in the respondent’s household. These factors may influence the adoption of health-seeking NPIs. Our logistic regression model estimates a linear regression defined as

(1) $$\mathrm{logit}\left(\pi_{i}\right)=x_{i}^{\prime }\beta ,$$

where the logit of probability $\pi_{i}$ is a linear function of the predictors; $x_{i}$ is a vector of covariates; β is a vector of coefficients, such that

(2) $$\frac{\pi_{i}}{1-\pi_{i}}=\exp\left\{x_{i}^{\prime }\beta \right\}$$

(3) $$\pi_{i}=\frac{\exp\left\{x_{i}^{\prime }\beta \right\}}{1+\exp\left\{x_{i}^{\prime }\beta \right\}}$$

(4) $$\mathrm{Health}-\mathrm{seeking}\;\mathrm{NPI}\;\left(\mathrm{mask}\;\mathrm{wearing}\right)=\{\begin{array}{cc} 1 & \mathrm{prob}\left(\pi_{i}\right)\\ 0 & \mathrm{prob}(1-\pi_{i}) \end{array}$$

We note that the left-hand side is in the familiar probability scale, while the right-hand side is a non-linear function of the predictors. Thus, our dependent variable mask wearing, as with other NPIs, takes the values 1 or zero with probabilities π_i_ and 1 − π_i_, respectively [25]. We estimated the logit using STATA to control for unobserved time effects as there is not sufficient statistics to condition fixed effects out of the likelihood in a logit function [26,27]. For a complete but non-mathematical treatment of logit models, this study draws from detailed expositions on logistic regression modeling [28,29] and with extensive applications and emphasis on model specification [26].

## 4. Results

This section provides details on the descriptive statistics of the variables and the results of the logistic regression estimations.

### Descriptive Statistics

Table 1 provides the means, standard deviation, and correlations of the variables used in the regression analysis. We estimated the variance inflation factors (VIF) for the variables and each model. We found that the range from 1.07 to 1.19 indicates the absence of multicollinearity. VIFs greater than 10 indicate model weakness due to multicollinearity [25]. The details of model adequacy are in Section 5.

**Figure 5:**
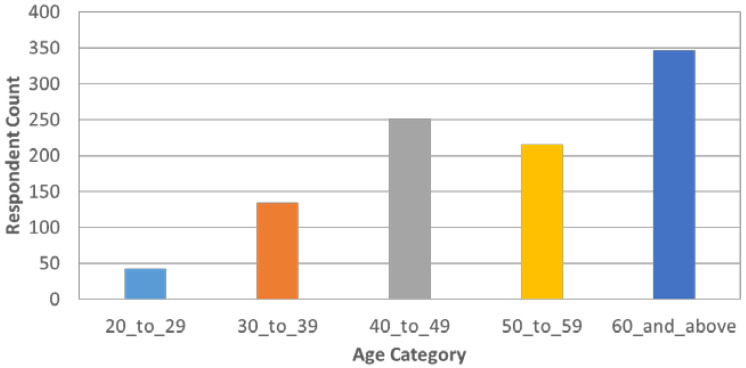
Age distribution of the respondents.

**Figure 6:**
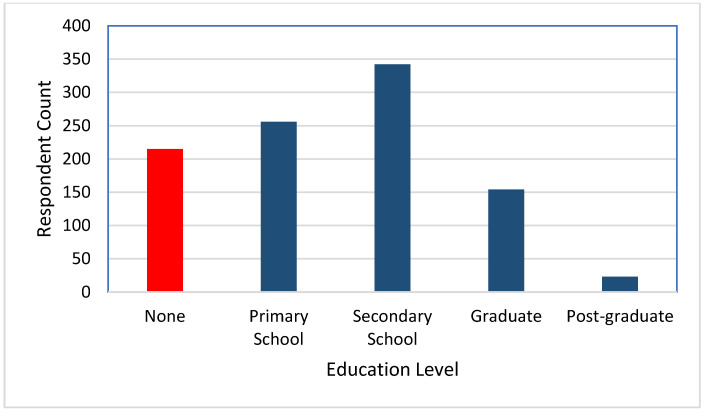
Education distribution of the respondents.

**Figure 7:**
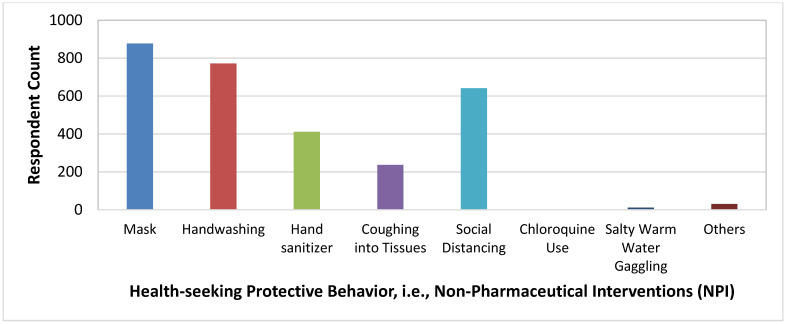
Distribution of the protective behaviors of the respondents.

**Figure 8:**
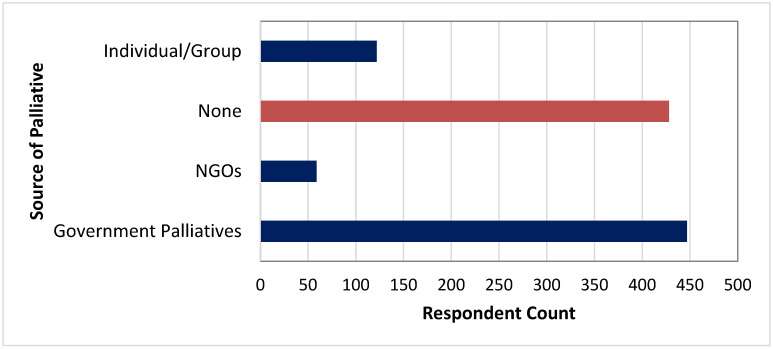
Palliatives distribution of the respondents.

**Figure 9:**
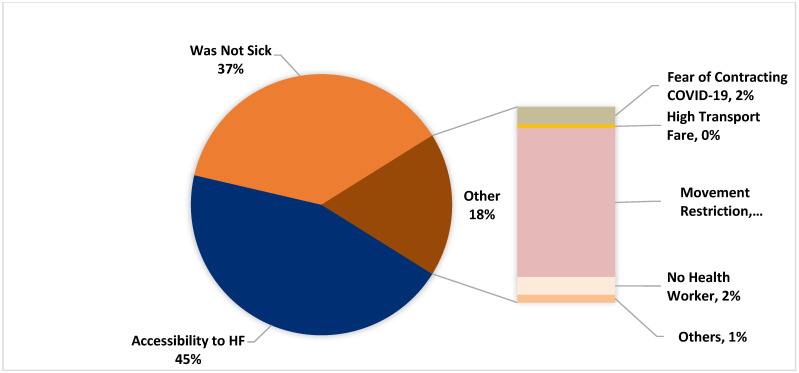
Distribution of access to health facility by the respondents.

Figure 5, Figure 6, Figure 7, Figure 8 and Figure 9 show the distributions of age, education, health-seeking NPIs, palliatives, and access to health facility.

Model 1 in Table 2 shows the control variables. The coefficient of the sex of the respondent is negative (β = −0.505) and statistically significant at 5% level, indicating that men are less likely to wear masks. Alternatively put, this result implies that women are more likely to wear masks. The statistical significance of this result underscores the consistency of the result with prior studies that indicate sex or gender differences in health-seeking behaviors [30].

The result shows that older people are less likely to wear masks with a relatively weak negative coefficient (β = −0.00037) that is not statistically significant. More interestingly, we observe that being educated (β = 0.547) and having students in the household (β = −0.569) are both positive coefficients and statistically significant at 5% level. Unexpectedly, we find that the larger the household size, the less likely they are to engage in mask wearing, though not statistically significant. In summary, the control variables indicate that females are more likely to wear masks than males, and being educated or having students in the household tend to promote mask wearing.

Model 2 tests the effect of awareness of COVID incidence on mask wearing. We postulated that awareness promotes health-seeking behavior. The result shows that the coefficient for COVID incident (β = 1.396) is positive and significant, indicating that opinions about COVID-19, especially the perceived risk of infection, increase with the social process of awareness, thereby enhancing the motivation and willingness to engage in health-seeking behavior through the NPI of mask wearing.

**Figure 10:**
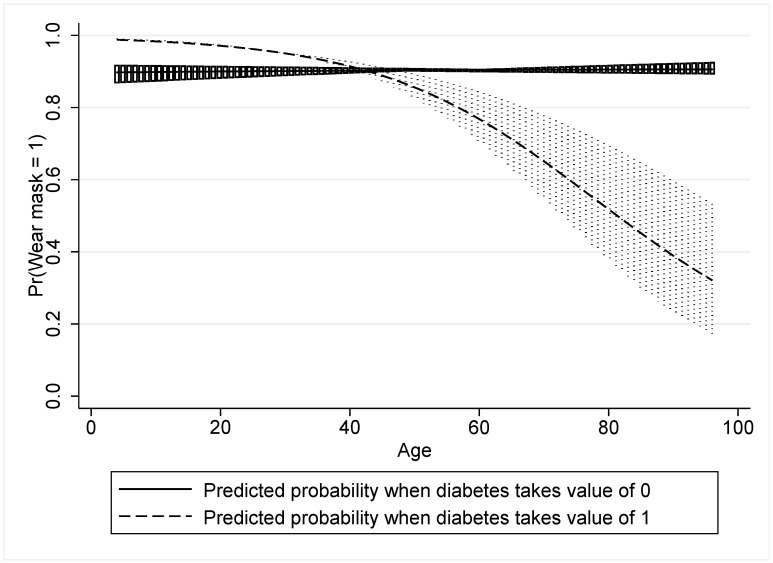
The interaction of respondent age and awareness of household diabetic under mask wearing.

Model 3 tests the awareness of the presence of an existing medical condition, such as having a diabetic in their households, and the positive coefficient (β = 0.015) shows that it does promote mask wearing as expected, but the result is not significant. The absence of significance for both age and a pre-existing morbidity led to the interaction of both respondent age and awareness of any diabetic in their households in Model 4. The result of a negative coefficient (β = −0.0423) was further explored in an interaction graph. Figure 10 shows quite an unexpected result that older people with diabetics in their households are less likely to wear masks than their counterparts who do not have diabetics in their households. More interesting is the observation of the converse, i.e., younger respondents with diabetic people in their households are more likely to wear masks than younger people without diabetics in their households.

The take-away message that is instructive from this interaction is that older people living in households without diabetic people are more likely to wear masks than their counterparts who live in households with diabetic people. Perhaps the psychological science interpretation is that mask wearing is seen as a protection from contracting COVID-19 by younger people with diabetics in their household, while the older respondents with diabetics in their household are hesitant to engage in this health-seeking intervention. 

Model 5 with a positive coefficient (β = 0.480) that is statistically significant at 5% level for health access demonstrates that access to a health facility encourages mask wearing as a health-seeking NPI. The rationale for this is that health facilities generally tend to promote health-seeking behaviors. Thus, the more access to clinics that community members have, the more likely they are to engage in protective behaviors.

Model 6 shows that the receipt of palliatives, independent of whether they were from the government or individuals or a non-governmental organization (NGO), has a strong and positive coefficient (β = 1.172) that is statistically significant at 1% level. This result is consistent with the value of aids that are provided in a pandemic, while providing some measures of relief also tends to enhance the adoption of protective behaviors. In sum, palliatives could be used as a mechanism to promote the adoption of NPIs.

The result in Model 7 with a negative coefficient (β = −0.597) that is significant at 5% level implies that the more access to food and supplies households have, the less likely mask wearing is. The rationale for this behavior is that with the abundance of food and essential supplies, the need to be exposed to causative agents is reduced, especially at the peak of the lockdown in the community.

## 5. Model Diagnostics

The bottom data in Table 2 shows that the Akaike Information Criterion (AIC) values are significantly high. This implies that the estimator of prediction error and, thereby, the relative quality of the statistical models for the respondent data is adequate. Likewise, the Bayesian Information Criterion (BIC) are even higher, indicating that the employed regression sufficiently provides a true model. Table 3 shows that the average Variance Inflation Factor (VIF) is 1.11. With a range of 1.07–1.19, the model is significantly void of multicollinearity, neither does any single variable dominate the explanatory effect. In general, VIFs greater than 10 indicate model weakness due to multicollinearity [25].

A cursory look at the R-squared values shows that they are relatively low. However, it is not uncommon for such low values in logistic regression and models that predict behavior. The more important observation is, though, that the noise portrayed by the low R-squared values, indicating a high-variability data, still captures the significant trends. In other words, the trend indicates that the predictors offer sufficient information about the response variable. While the regression coefficients estimate the trends, the R-squared values reflect the scatter around the regression line [31]. In essence, the influence of the significant variables is independent of the strength of the R-squared values. In logistic regression with binary or ordinal dependent variables, less reliance should be placed on R-squared or pseudo-R-squared statistics [32,33].

## 6. Robustness Tests

We estimated several robustness tests. We re-estimated the full Model 7 in Table 4 without the constant terms to ascertain that our prior results stand [34,35] and are even improved with some variables becoming significant.

The results are largely consistent with those in the main models where the constant term is a part of the predictors. However, we note here that not only do the prior results stand, but we also now have age and household size becoming significant at 5% and 1% levels, respectively. Though the values of the coefficients changed marginally, this only further reiterates that the properties of the logistic model imply that the magnitude of the effects may not be inferred from the coefficients, but only the sign and statistical significance of the variables [36,37].

We report a second and major test of robustness by repeating Model 7 from Table 2 for hand washing, use of hand sanitizer, and social distancing. The results shown in Table 5 indicate that the psychosocial responses, significantly, remain consistent across these other NPIs as we had for mask wearing. Of importance are the changes in model outcomes for social distancing where education became negative and significant (β = −0.581) at 1% and having a student in the household flipped to being negative. More importantly is the health access becoming significant as it was for mask wearing (β = 0.423) at 1%. Otherwise, we see that the interaction of household diabetics and respondent age also became positive and significant (β = 0.631) at 0.1%.

**Figure 11:**
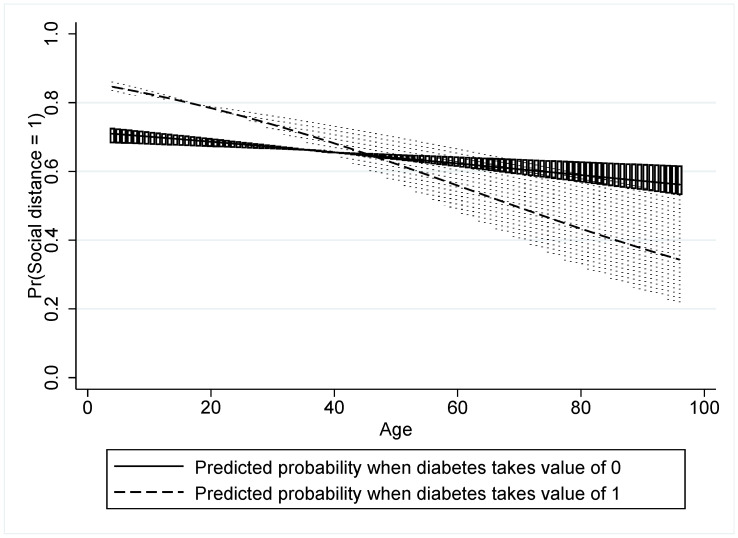
The interaction of age and diabetics in household under social distancing.

We graph this interaction in Figure 11 to show that the behavior is similar to the results under mask wearing, i.e., for the key NPI prescriptions, older people without diabetics in their household are more likely to social distance, just as they are more likely to wear masks, than their counterparts who have diabetics in their households. The response of hand washing and the use of hand sanitizer are significantly aligned with the mask wearing and social distancing results except for the limited level of significance for the variables in the two models. Lastly, we note that the AIC and BIC metrics are consistent with the earlier values for mask wearing, demonstrating model adequacy.

## 7. Discussion

Our results indicate that there are variations in how these parameters influence specific mitigation practices. Specifically, we find that: (i) women are more likely to engage in mask wearing, hand washing, and use of hand sanitizers than men, (ii) the provision of palliatives and access to family supplies enhances the adoption of these community mitigation interventions. While these psychosocial factors influence the adoption of the COVID-19 NPI practices in different and often surprising ways, it is incisive to note there is a level of statistical coherence in the effects of these behaviors. For example, the outcome that younger respondents with diabetics in their households are more likely to engage in mask wearing or social distancing than their peers who do not have diabetics in their households underscores the relative manner in which these health-seeking behaviors are interpreted. Integrating these observations, we note that mask wearing, or any other NPI, has levels of discomfort, especially in humid weather, such that the notion of wearing a protective material over the nose and mouth may limit not just verbal but also nonverbal communication cues [38]. Nonetheless, the effort to mitigate the spread of the pandemic takes precedence over the individual levels of discomfort. In addition, the hesitancy toward mask wearing induced by concerns about reduced oxygen saturation or carbon dioxide accumulation has not been supported by examined data.

This study sheds light on the crafting of healthcare policies for pandemic mitigation in three ways. First, we note that awareness of COVID-19 incidence or death is a catalyst for community adoption of the NPIs. Thus, it is important for healthcare policy to be inclusive of information dissemination that is effective on updates of incidence or death rates because awareness as a psychosocial construct may provide inherent motivation to embrace community mitigation practices. The need for communication resilience is even stronger in the advent of a disaster or pandemic [39]. This also helps to examine how well existing processes and structures were mobilized during the pandemic. Perhaps customized efforts might be targeted at a specific gender to achieve risk-minimizing behavioral changes.

Second, the provision of economic palliative measures could serve as the invisible incentive to promote the adoption of NPIs. Combining these two implications for policy leads to a sweet spot, implying that it is not enough to merely broadcast community mitigation measures, it is also crucial to put in place responsive initiatives in the form of palliatives to support the community. Third, the overall community benefit of engaging in the adoption of NPIs is a derivative of the psychosocial parameters to limit the propagation media, i.e., air-borne or contaminated surfaces, by which the causative SARS-CoV-2 virus spreads. We draw a parallel between the achievement of herd immunity for vaccination and the more the community embraces the NPIs as prerequisites to eradicating the COVID-19. More importantly, for countries such as Nigeria still grappling with improving their vaccine supply chains [40,41], the best option for mitigating the pandemic may very well be through the widespread adoption of NPIs. 

This paper makes three key contributions. First, enhancing community self-efficacy to predict the intention to adopt health-seeking behaviors through NPIs is influenced at different scales by a menu of socioeconomic indicators. Second, repeated public awareness of disease spread and progression during a pandemic in the community is a prerequisite to improve community mitigation practices. Third, these observations combine to inform healthcare policymaking in ways that are not always readily observable.

Our study has several limitations that provide opportunities for future research. First, though our results are statistically significant in the context of a low- and middle-income country such as Nigeria, it is left to be seen to what extent these findings hold when examined in a developed country. Second, the study left it to be ascertained the impact of the time interval in which the data was collected. Perhaps a continuous data collection effort spanning a much longer time interval could further emphasize these observations. Third, the spread of the pandemic in Nigeria is not as pronounced as we have observed in countries such as Italy, India, or even the U.S. Consequently, it is an open question as to what extent these results are confirmed in countries with significant rates of infections.

## 8. Conclusions

A crucial finding is that health-seeking NPI behaviors are statistically significantly affected in different ways by the menu of socioeconomic indicators. For example, the level of social awareness of the pandemic’s incidence and mortality rates influences the extent to which communities embrace health-seeking NPIs. The control for age, sex, education, and household size indicates that there is intersectionality of how these factors influence specific mitigation practices. We find that women are more likely to engage in mask wearing, hand washing, and the use of hand sanitizers and tissues than men, and the provision of palliatives and access to family supplies significantly enhances community mitigation. These findings help to reinforce the complexity behind the stipulation of the NPIs and some of the overarching unintended risk outcomes that have been reported to overshadow the interventions. This study underlines significant implications for pandemic mitigation policy, i.e., providing regular awareness to the community on the progress and intensity of the pandemic combined with the provision of economic palliative measures could serve as catalysts for enhancing the adoption of community mitigation practices that might, in turn, limit the spread of the pandemic. The prescription for the government of Nigeria (and other low- and middle-income countries) is that to minimize morbidity and mortality, as well as the social and economic impact of COVID-19, it is not enough to merely broadcast community mitigation measures, it is also crucial to put in place responsive initiatives such as routine information updates on disease progression and the provision of relief supplies. Theoretically, we contribute to the literature on pandemic mitigation policies by shedding light on the intricacies surrounding the impacts of psychosocial metrics on the adoption of mitigation initiatives.

## Figures and Tables

**Table 1:** Descriptive statistics: Means and correlation matrix. Note: Std. Dev. stands for standard deviation.

Variables	Mean	Std Dev.	Mask	Sex	Age	Education	Household Size	Student in Household	COVID Incident	Diabetes	Health Access	Palliatives	Food & Supplies
Mask	0.89	0.32	1										
Sex	0.62	0.49	–0.08 ***	1									
Age	42.55	16.08	–0.04	0.21 ***	1								
Education	0.78	0.41	0.10 ***	0.01	–0.23 ***	1							
Household Size	11.57	9.00	–0.08 **	0.18 ***	0.12 ***	–0.15 ***	1						
Student in HH	0.89	0.32	0.07 **	–0.01	0.02	0.17 ***	0.19 ***	1					
COVID Incident	0.10	0.30	0.08 **	0.04	–0.01	0.06 **	0.14 ***	0.004	1				
Diabetes	0.08	0.27	–0.01	0.09 ***	0.05 *	–0.03	0.22 ***	0.02	0.16 ***	1			
Health Access	0.45	0.50	0.09 ***	0.07 **	0.12 ***	0.11 ***	0.002	0.08 ***	0.09 ***	0.04	1		
Palliatives	0.48	0.50	0.14 ***	0.13 ***	0.08 ***	–0.11 ***	0.1 ***	–0.06 **	0.08 **	0.03	0.07 **	1	
Food & Supplies	0.73	0.45	–0.08 **	0.021	–0.14 ***	–0.04	0.04	0.05 *	0.06 **	0.09 ***	–0.16 ***	–0.01	1

Standard errors in parentheses, *** *p* < 0.01, ** *p* < 0.05, * *p* < 0.10: *p*-value is the probability of obtaining test results at least as extreme as the results actually observed, under the assumption that the null hypothesis is correct.

**Table 2:** Random effects logistic model: DV = mask wearing (1) or not wearing mask (0). Note—Hh: Household.

	Model 1	Model 2	Model 3	Model 4	Model 5	Model 6	Model 7
Sex	–0.505 *	–0.515 *	–0.515 *	–0.519 *	–0.523 *	–0.658 **	–0.624 **
	(0.23)	(0.23)	(0.23)	(0.23)	(0.23)	(0.24)	(0.23)
Age	–0.000372	–0.000361	–0.00037	0.00329	–0.00237	–0.00369	–0.00607
	(0.01)	(0.01)	(0.01)	(0.01)	(0.01)	(0.01)	(0.01)
Education	0.547 *	0.487 *	0.487 *	0.490 *	0.408	0.544 *	0.505 *
	(0.23)	(0.24)	(0.24)	(0.24)	(0.24)	(0.25)	(0.25)
Household Size	–0.0184	–0.0250 *	–0.0251 *	–0.0246 *	–0.0247 *	–0.0314 **	–0.0321 **
	(0.01)	(0.01)	(0.01)	(0.01)	(0.01)	(0.01)	(0.01)
Students in Household	0.569 *	0.648 *	0.649 *	0.619 *	0.607 *	0.737 *	0.814 **
	(0.29)	(0.29)	(0.29)	(0.29)	(0.29)	(0.30)	(0.31)
COVID Incident		1.396 **	1.393 **	1.379 *	1.316 *	1.200 *	1.242 *
		(0.54)	(0.54)	(0.54)	(0.54)	(0.54)	(0.54)
Diabetes in Hh			0.015	2.152	0.0132	0.102	0.198
			(0.39)	(1.31)	(0.39)	(0.40)	(0.41)
Diabetes in Hh X Age				–0.0423			
				(0.02)			
Health Access					0.480 *	0.476 *	0.377
					(0.22)	(0.22)	(0.23)
Palliatives						1.172 ***	1.160 ***
						(0.23)	(0.23)
Food and Supplies							–0.597 *
							(0.28)
Constant	1.739 ***	1.711 ***	1.711 ***	1.571 ***	1.707 ***	1.278 **	1.821 ***
	(0.42)	(0.42)	(0.42)	(0.43)	(0.42)	(0.44)	(0.51)
AIC	692.7	684.8	686.8	685.3	683.9	658	654.9
BIC	722	719.1	726	729.3	728	707	708.8
N	990	990	990	990	990	990	990

Standard errors in parentheses, * *p* < 0.05, ** *p* < 0.01, *** *p* < 0.001; *p*-value is the probability of obtaining test results at least as extreme as the results actually observed, under the assumption that the null hypothesis is correct.

**Table 3:** Model diagnostic test.

Variable	VIF	Tolerance	R–Squared
Mask	1.07	0.93	0.07
Sex	1.11	0.90	0.10
Age	1.17	0.86	0.14
Education	1.18	0.85	0.15
Household Size	1.19	0.84	0.16
Student in Household	1.11	0.90	0.10
COVID Incident	1.07	0.94	0.06
Diabetes	1.08	0.92	0.08
Health Access	1.08	0.92	0.08
Palliatives	1.08	0.93	0.07
Food and Supplies	1.08	0.93	0.07
Mean VIF	1.11		

**Table 4:** Robustness test: Model without constant term. Note—Hh stands for Household (Standard errors in parentheses, *** *p* < 0.01, ** *p* < 0.05, * *p* < 0.10).

Mask Wearing	Coefficient	St. Err.	t-Value	*p*-Value	[95% Conf. Interval]		Sig
Sex	–0.495	0.225	–2.200	0.028	–0.935	–0.055	**
Age	0.009	0.005	1.680	0.092	–0.002	0.020	*
Education	0.901	0.212	4.250	0.000	0.485	1.318	***
Household Size	–0.027	0.012	–2.33	0.020	–0.05	–0.004	**
Student in Hh	1.100	0.274	4.010	0.000	0.562	1.637	***
COVID Incident	1.230	0.544	2.260	0.024	0.164	2.296	**
Diabetes	0.115	0.407	0.280	0.778	–0.682	0.912	
Health Access	0.439	0.226	1.950	0.052	–0.003	0.881	*
Palliatives	1.307	0.231	5.640	0.000	0.853	1.760	***
Food and Supplies	–0.127	0.222	–0.570	0.568	–0.561	0.308	
Number of obs	990						
Chi–square	350						
Prob > chi2	0						
Akaike crit. (AIC)	667						

**Table 5:** Robustness check of the full model using other NPIs as dependent variable (Standard errors in parentheses, *** *p* < 0.01, ** *p* < 0.05, * *p* < 0.10).

	Hand Washing	Hand Sanitizer	Social Distancing
Sex	–0.789 ***	–0.540 ***	0.098
	(0.18)	(0.15)	(0.15)
Age	0.01	–0.016 ***	–0.006
	(0.01)	(0.01)	(0.00)
Education	0.463 *	0.149	–0.581 **
	(0.20)	(0.18)	(0.19)
Household Size	–0.001	0.030 ***	–0.012
	(0.01)	(0.01)	(0.01)
Students in Household	–0.023	–0.006	–0.267
	(0.25)	(0.23)	(0.24)
COVID Incident	0.517	0.565 *	0.047
	(0.31)	(0.23)	(0.24)
Diabetes	0.371	0.434	0.288
	(0.34)	(0.26)	(0.28)
Diabetes X Age	–0.014	–0.179	0.631 ***
	(0.17)	(0.14)	(0.15)
Health Access	0.152	0.037	0.423 **
	(0.16)	(0.14)	(0.14)
Palliatives	0.651 ***	0.725 ***	0.553 ***
	(0.17)	(0.16)	(0.16)
Food & Supplies	0.457	–0.384	0.772 *
	(0.39)	(0.35)	(0.36)
AIC	1023.1	1277.6	1253
BIC	1077	1331.5	1306.8
N	990	990	990

## Data Availability

All data generated or analyzed during this study are included in this published article and available from the corresponding author on reasonable request.

## Abbreviations

AIC	Akaike Information Criterion
BIC	Bayesian Information Criterion
COMCiRIN	Coordinating and Mobilizing Civil Society Response in Nigeria
COVID-19	SARS-CoV-2 pandemic
CSO	Civil Society Organizations
FCT	Federal Capital Territory
LGA	Local Government Areas
LQAS	Lots Quality Assessment Survey
NPI	Non-Pharmaceutical Intervention
ODK	Open Data Kit
PPS	Probability of Proportional to Sample size
PTF	Presidential Task Force
SPHCDA	State Primary Health Care Development Agency
VIF	Variance Inflation Factors
WAVA	Women Advocates for Vaccine Access
WHO	World Health Organization
